# Revision and phylogeny of the genus *Loxoneptera* Hampson, 1896 (Lepidoptera, Crambidae, Pyraustinae), based on morphology and molecular data

**DOI:** 10.3897/zookeys.1036.63814

**Published:** 2021-05-05

**Authors:** Lanbin Xiang, Kai Chen, Dandan Zhang

**Affiliations:** 1 School of Life Sciences, Sun Yat-sen University, Guangzhou, Guangdong 510275, China; 2 School of Ecology, Sun Yat-sen University, Guangzhou, Guangdong 510275, China; 3 School of Life Sciences, Jiaying University, Meizhou, 514015, China

**Keywords:** *
Calamochrous
*, China, molecular phylogeny, new combinations, new species

## Abstract

The genus *Loxoneptera* Hampson, 1896 is revised based on external appearance and genitalia. It is comprised of eleven species, of which three are described as new species from China: *L. crassiuncata* Chen & Zhang, **sp. nov.**, *L. triangularis* Chen & Zhang, **sp. nov.**, and *L. rectacerosa* Chen & Zhang, **sp. nov.**; six species are proposed as new combinations: *L. carnealis* (Swinhoe, 1895), **comb. nov.**, *L. medialis* (Caradja, 1925), **comb. nov.**, *L. pentasaris* (Meyrick, 1932), **comb. nov.**, *L. bipunctalis* (Hampson, 1912), **comb. nov.**, *L. brevipalpis* (Snellen, 1890), **comb. nov.**, and *L. dichroma* (Moore, 1888), **comb. nov.** A new replacement name, *L. hampsoni* Chen & Zhang, **nom. nov.**, is proposed for *L. carnealis* Hampson, 1896, the type species of the genus, because it is a secondary homonym of *L. carnealis* (Swinhoe, 1895), **comb. nov.** External characters and genitalia morphology of all species are figured. Nucleotide sequences of COI, 16S rRNA, 28S rRNA, and EF-1α were used for the molecular analysis and phylogeny of *Loxoneptera* species.

## Introduction

The genus *Loxoneptera* was established as a monotypic genus by Hampson (1896), based on *L. carnealis* Hampson, 1896 from Sikkim and Assam. Subsequently, Swinhoe (1906) described a new species, *L. albicostalis*, from Padang, Sumatra, mainly based on appearance of the wings. The genus was not investigated again until Chen et al. (2018) who, for the first time, recorded these two species in China (see also Nuss et al. 2003–2021). They noted that *Loxoneptera* was paraphyletic, with respect to two species of *Calamochrous* Lederer, 1863, i.e., *C. carnealis* (Swinhoe, 1895) and *C. medialis* Caradja, 1925, appeared as terminal lineages within *Loxoneptera* clade based on a molecular phylogenetic analysis. But *C. carnealis* and *C. medialis* were not transferred to *Loxoneptera* in their study.

Within the additional Chinese specimens collected, three undescribed species of *Loxoneptera* were recognised. Moreover, a few species of *Calamochrous* and *Anania* Hübner, 1823 were found to be congeneric with species of *Loxoneptera*. The aim of this study is to diagnose *Loxoneptera* based on external and genital characters, to clarify the species included in the genus, and to provide a preliminary phylogenetic hypothesis based on selected genetic markers.

## Materials and methods

The material studied, including the types of the newly described species, are all deposited at the Museum of Biology, Sun Yat-sen University, China (**SYSBM**) except those stored in the following institutions: Insect Collection of the College of Life Sciences, Nankai University, China (**NKU**), Forest Canopy Ecology Lab, Yunnan, China (**FCEL**), “Grigore Antipa” National Museum of Natural History, Romania (**MGAB**), and Natural History Museum, London, United Kingdom (**NHMUK**). Slides of genitalic dissections were prepared according to Robinson (1976) and Li and Zheng (1996), with some modifications. Genitalia terminology follow Klots (1970), Munroe (1976), Maes (1995), and Kristensen (2003). Images of the adults were taken using a Canon EOS 60D camera provided with a Canon 100 mm macro lens; the genitalia images were taken using Zeiss Axio Scope.A1 in combination with a Zeiss AxioCam camera and the Axio Vision SE64 program on a Windows PC; source images were then aligned and stacked on Helicon Focus to obtain a fully sharpened composite image. All images were edited using Adobe Photoshop SC5.

Ten species in four genera were included in the molecular phylogenetic analyses (Table 1). *Euclasta stoetzneri* (Caradja, 1927) was chosen as the outgroup because it has been inferred as sister-group of Pyraustini and Portentomorphini in Pyraustinae (Mally et al. 2019). One species of *Sclerocona* Meyrick, 1890 and three species of *Eumorphobotys* Munroe & Mutuura, 1969 were also included as related genera to *Loxoneptera* according to Chen et al. (2018). Total DNA was extracted from two legs, and sometimes from the abdomen of the dry specimens using the TIANGEN DNA extraction kit following the manufacturer’s instructions. The nucleotide sequences of two mitochondrial genes, cytochrome c oxidase subunit I (COI) and 16S ribosomal RNA (16S rRNA), and two nuclear genes, 28S ribosomal RNA (28S rRNA) and Elongation factor-1 alpha (EF-1α) were selected for study. Primers used in this study and all PCRs performed are the same as in Zhang et al. (2020). PCR products were confirmed with 1.5% agarose gel electrophoresis in TAE buffer, then were purified and direct-sequenced at Majorbio Bio-pharm Technology Co., Ltd (Guangzhou), utilising the same primers used for PCR amplification.

**Table 1.** Species sampled for the molecular phylogenetic analysis.

Genus	Species	Voucher	Locality	GenBank accession number				References
				COI	16S	EF-1α	28S	
* Eumorphobotys *	* eumorphalis *	SYSULEP0046	Fujian	MG739574	MG739586	MG739598	MG739609	Chen et al. 2018
		SYSULEP0047	Fujian	MG739575	MG739587	MG739599	MG739610	Chen et al. 2018
	* concavuncus *	SYSULEP0042	Yunnan	MG739571	MG739583	MG739595	MG739606	Chen et al. 2018
		SYSULEP0175	Guangxi	MG739581	MG739593	MG739604	MG739616	Chen et al. 2018
	* horakae *	SYSULEP0043	Sichuan	MG739572	MG739584	MG739596	MG739607	Chen et al. 2018
		SYSULEP0172	Sichuan	MG739580	MG739592	N/A	MG739615	Chen et al. 2018
* Loxoneptera *	* hampsoni *	SYSULEP0166	Hainan	MG739579	MG739591	MG739603	MG739614	Chen et al. 2018
		SYSULEP0174	Hainan	MW736545	MW736550	MW736555	MW728364	Present study
	* albicostalis *	SYSULEP0162	Yunnan	MG739578	MG739590	MG739602	MG739613	Chen et al. 2018
	* medialis *	SYSULEP0096	Hainan	MG739576	MG739588	MG739600	MG739611	Chen et al. 2018
		SYSULEP0171	Guangdong	MW736546	MW736551	MW736556	MW728365	Present study
		SYSULEP0173	Guangdong	MW736547	MW736552	N/A	N/A	Present study
	* rectacerosa *	SYSULEP0170	Yunnan	MW736548	MW736553	N/A	N/A	Present study
	* carnealis *	SYSULEP0044	Guizhou	MG739573	MG739585	MG739597	MG739608	Chen et al. 2018
		SYSULEP0186	Yunnan	MW736549	MW736554	MW736557	MW728366	Present study
* Sclerocona *	* acutella *	SYSULEP0152	Macau	MG739577	MG739589	MG739601	MG739612	Chen et al. 2018
* Euclasta *	* stoetzneri *	SYSULEP0334	Shannxi	MT738696	MT734412	MT724335	MT734404	Zhang et al. 2020

The sequences were aligned using Clustal W (Thompson et al. 1994) in MEGA 6 (Tamura et al. 2013) with default settings. The aligned matrix was corrected by eye. Gaps were treated as missing data. Phylogenetic analyses were inferred using Bayesian inference (BI) method in MrBayes 3.2.6 (Ronquist et al. 2012) and maximum likelihood (ML) in RAxML 8.2.10 (Stamatakis 2014). BI analysis was run with independent parameters for the COI, the 16S rRNA and 28S rRNA gene partitions under the GTR + G model, the EF-1α gene partition under the GTR + G + I model, as suggested by jModelTest 0.1.1 (Posada 2008). Two independent runs, each with four Markov Chain Monte Carlo (MCMC) simulations, were performed for 20 million generations sampled every 1000^th^ generation. The first 25% trees were discarded as burn-in, and posterior probabilities (PP) were determined from remaining trees. ML analysis was executed under the GTR + G model for all gene partitions and with 1000 iterations for the bootstrap test. The pairwise Kimura 2-Parameter ( K2P) distances between species were calculated from the COI gene using MEGA 6 (Tamura et al. 2013).

## Results

### Phylogenetic relationships

**Figure 1.**
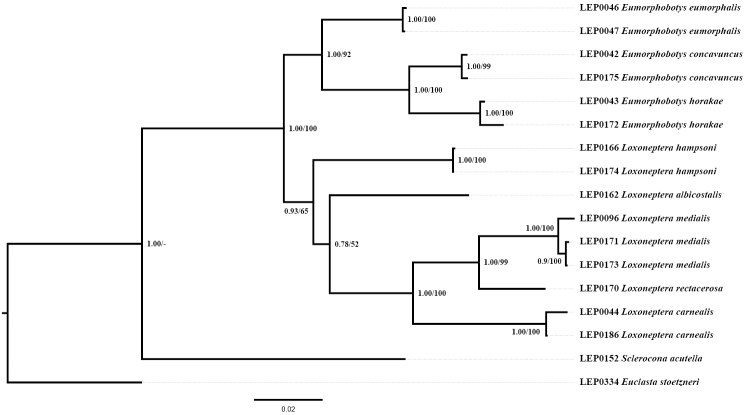
Phylogenetic hypothesis inferred from Bayesian inference. Numbers on branches indicate Bayesian posterior probabilities and ML bootstrap values, respectively.

The concatenated dataset of four genes consisted of 2511 nucleotide positions (658 for COI, 463 for 16S rRNA, 619 for 28S rRNA, and 771 for EF-1α). Both BI and ML analyses of the concatenated dataset inferred congruent topologies with only subtle differences in posterior probability and bootstrap values probability (Fig. 1). The monophyly of *Loxoneptera* is strongly supported in BI but weakly supported in ML (PP = 0.93, BS = 65). *Eumorphobotys* is in a sister group position to *Loxoneptera* with robust support (PP = 1.00, BS = 100).

The results of the current phylogenetic analyses support that the undescribed species (here named as *L. rectacerosa* sp. nov.) should be placed in *Loxoneptera*, and that *L. carnealis* (Swinhoe, 1895) comb. nov. and *L. medialis* (Caradja, 1925) comb. nov. should be transferred from *Calamochrous* Lederer, 1863 to *Loxoneptera*. Within the genus, *L. medialis* + *L. rectacerosa* form a sister group with robust support (PP = 1.00, BS = 99), while *L. carnealis* is the sister group to *L. medialis* + *L. rectacerosa* (PP = 1.00, BS = 100). *Loxoneptera albicostalis* is associated with the clade *L. carnealis* + (*L. medialis* + *L. rectacerosa*), although with relatively low support (PP = 0.78, BS = 52). *Loxoneptera hampsoni* is the first-diverging species with strong support in the BI analysis (PP = 0.93), but with relatively low support in the ML analysis (BS = 65).

Pairwise distances of the barcoding region (COI) are given in Table 2. The genetic distances between *Loxoneptera* and other genera range from 7.7% (*Eumorphobotys*) to 12.4% (*Sclerocona*). Interspecific genetic distances within *Loxoneptera* range from 4.2% (*L. medialis* to *L. rectacerosa*) to 11.2% (*L. hampsoni* to *L. carnealis*), while intraspecific genetic distances in *Loxoneptera* range from 0 % (*L. medialis*) to 0.3% (*L. carnealis*).

**Table 2.** Pairwise distance of the COI barcoding region based on Kimura-2-parameter model.

		1	2	3	4	5	6	7	8	9	10	11	12	13	14	15	16
1	LEP0046 *Eumorphobotys eumorphalis*																
2	LEP0047 *Eumorphobotys eumorphalis*	0.000															
3	LEP0042 *Eumorphobotys concavuncus*	0.072	0.072														
4	LEP0175 *Eumorphobotys concavuncus*	0.070	0.070	0.006													
5	LEP0043 *Eumorphobotys horakae*	0.078	0.078	0.068	0.066												
6	LEP0172 *Eumorphobotys horakae*	0.074	0.074	0.068	0.066	0.003											
7	LEP0166 *Loxoneptera hampsoni*	0.096	0.096	0.112	0.106	0.119	0.119										
8	LEP0174 *Loxoneptera hampsoni*	0.096	0.096	0.112	0.106	0.119	0.119	0.000									
9	LEP0162 *Loxoneptera albicostalis*	0.087	0.087	0.091	0.091	0.107	0.103	0.087	0.087								
10	LEP0096 *Loxoneptera medialis*	0.087	0.087	0.094	0.092	0.099	0.098	0.090	0.090	0.077							
11	LEP0171 *Loxoneptera medialis*	0.087	0.087	0.092	0.090	0.099	0.097	0.092	0.092	0.077	0.002						
12	LEP0173 *Loxoneptera medialis*	0.087	0.087	0.092	0.090	0.099	0.097	0.092	0.092	0.077	0.002	0.000					
13	LEP0170 *Loxoneptera rectacerosa*	0.077	0.077	0.089	0.090	0.101	0.099	0.099	0.099	0.080	0.044	0.042	0.042				
14	LEP0044 *Loxoneptera carnealis*	0.101	0.101	0.103	0.107	0.118	0.118	0.112	0.112	0.080	0.067	0.065	0.065	0.082			
15	LEP0186 *Loxoneptera carnealis*	0.101	0.101	0.103	0.105	0.117	0.117	0.108	0.108	0.077	0.064	0.062	0.062	0.079	0.003		
16	LEP0152 *Sclerocona acutella*	0.099	0.099	0.113	0.113	0.119	0.119	0.108	0.108	0.108	0.106	0.104	0.104	0.102	0.124	0.124	
17	LEP0334 *Euclasta stoetzneri*	0.106	0.106	0.118	0.118	0.135	0.135	0.111	0.111	0.118	0.106	0.104	0.104	0.101	0.115	0.115	0.118

### Taxonomic account

#### Loxoneptera

Taxon classificationAnimaliaLepidopteraCrambidae

Hampson, 1896

B41A37D0-7163-5A10-A5AC-A98792CB6B2E

Loxoneptera Hampson, 1896: 405. Type species: Loxoneptera carnealis Hampson, 1896, by original designation.

##### Diagnosis.

In external appearance, the species of *Loxoneptera* are similar to species of *Eumorphobotys* Munroe & Mutuura, 1969 in the long and porrect labial palpus, the usually concolorous wings with no obvious pattern and the straight termen of forewing, but can be best distinguished by the triangular uncus, the rod-shaped dorsal projection of transtilla bearing long and thick hair at the apex, and the hook-shaped ventral sella in the male genitalia. In the female genitalia, the ductus bursae of *Loxoneptera* is shorter and stouter than that of *Eumorphobotys*. These two genera are also different in the shape of the signum, if present a nearly rhomboid signum with connected carina, or reduced into a keel-like carina in *Loxoneptera*, and a narrowly rhomboid signum with carina interrupted in *Eumorphobotys*. Eighth sternite in males of *Loxoneptera* is slightly sclerotised, with two slender and sclerotised anterolateral processes.

##### Description.

***Head*.** Frons oblique, slightly protruding. Vertex with moderately raised scales projecting between antennae. Labial palpus ~ 2–2.5 × eye diameter; second segment obliquely upward, third segment long and porrect. Maxillary palpus small. ***Thorax*.** Legs unmodified usually, outer spur 1/3 to 1/2 the length of inner spur, sometimes outer spur minute. ***Wings*.** Forewing elongated triangular, termen obliquely straight to slightly curved; discal cell ~ 1/2 length of wing, R_1_ from ~ 3/4 of anterior margin of cell, R_3_ and R_4_ stalked to more than half of R_4_, R_5_ free from anterior angle of cell, parallel to stalked R_3_+R_4_ at base, then diverging, discocellular veins concavely curved, M_1_ close to R_5_ at base, free from discocellular veins and close to anterior angle of cell, M_2_, M_3_ and CuA_1_ from posterior angle of cell, CuA_2_ from 4/5 of the posterior margin of cell, 1A faintly sinuate to tornus; 2A forming complete loop and distally recurved before joining 1A; usually only with orbicular and reniform stigmata, sometimes no pattern. Hindwing fan-shaped, termen rounded; discal cell less than half length of wing, Sc+R_1_ and Rs anastomosed to half of Rs, discocellulars concave, M_2_, M_3_ and CuA_1_ from posterior angle of discal cell, CuA_2_ from 4/5 of the posterior margin of cell; without obviously spot. ***Abdomen*.** Eighth sternite in male with two slender and sclerotised anterolateral processes, pointed or slightly stout (Fig. 14).

***Male genitalia.*** Uncus triangular, glabrous or with few hair-like setae. Tegumen trapezoid. Saccus nearly triangular. Transtilla with developed ventral process, extending a rod-shaped projection dorsad, usually long, curved, and slender, and terminal part with many long hairs. Valva tongue-shaped; dorsal sella membranous, ventral sella usually with a hook-shaped, strongly sclerotised process, dorso-distal sella presented as a sclerite and usually extended as a long, hook-shaped, sclerotised process; editum absent or not obvious; sacculus broad. Juxta with basal part rivet-shaped, remainder usually with two long and slender bifid arms. Phallus tubular, vesica with spine-shaped cornuti and sometimes deciduous cornuti.

***Female genitalia.*** Ovipositor lobes flat, densely setose. Anterior apophyses longer than posterior apophyses. Antrum sclerotised, cup-shaped or bowl-shaped; colliculum well developed and sclerotised; ductus seminalis entering near anterior end of colliculum; ductus bursae short and stout, almost as long as length of corpus bursae; corpus bursae oval, appendix bursae oval or absent, signum nearly rhomboid, with a carina not interrupted in middle, sometimes signum reduced into a carina, sometimes absent.

##### Distribution.

China, India, Indonesia, Malaysia.

### Key to species of *Loxoneptera*

**Table:** 

1	Forewing reddish brown; hindwing black-brown in male, with a triangular patch presented near the posterior angle of cell	**2**
–	Forewing colour paler, not reddish brown; hindwing pale yellow, triangular patch absent	**3**
2	Costal band of forewing white, fringe white and with basal 1/4 black-brown (Fig. 4), a small triangular indentation presented on the 1/3 of posterior margin in male; ventral sella with a long hook-shaped process; vesica without cornutus (Fig. 16)	***L. albicostalis***
–	Costal band of forewing brown, fringe pale yellow and with basal half black-brown (Fig. 5), posterior margin of male smooth; ventral sella with a relatively short and stick-like process; vesica with a horn-shaped, strongly sclerotised cornutus apically (Fig. 17)	***L. crassiuncata***
3	Forewing with pale yellow stripes between veins, posterior margin with a small triangular indentation and a group of black-brown scales in male (Fig. 2); juxta medially concave inwardly (Fig. 15)	***L. hampsoni***
–	Forewing without pale yellow stripe between veins, posterior margin arc-shaped, without indentation and a group of black-brown scales in male; juxta normal	**4**
4	Distal part of phallus with a long and pointed spine, longer than the length of phallus (Fig. 18)	***L. carnealis***
–	Distal part of phallus without spine, or spine shorter than the length of phallus	**5**
5	Distal part of juxta with a strongly sclerotised and narrowly triangular process (Fig. 19)	***L. triangularis***
–	Distal part of juxta without process	**6**
6	Distal end of phallus densely decorated with short spines (Fig. 22)	***L. pentasaris***
–	Distal end of phallus not decorated with short spines	**7**
7	Dorsal margin of valva forming a break angle subapically (Fig. 20)	***L. rectacerosa***
–	Dorsal margin of valva without break angle	**8**
8	Dorsal margin of valva convex, dorso-distal sella extended outwards and not beyond the end of valva (Fig. 21)	***L. medialis***
–	Dorsal margin of valva somewhat concave, dorso-distal sella extended ventrad and beyond the ventral margin of valva	**9**
9	Forewing with a stripe along posterior margin of cell (Fig. 13); ventral-distal wall of phallus weakly sclerotised and obliquely extended into a process (Fig. 25)	***L. dichroma***
–	Forewing absent stripe on posterior margin of cell; wall of phallus not sclerotised	**10**
10	Distal part of phallus with a heavily sclerotised, spiny and thumb-shaped cornutus (Fig. 24)	***L. brevipalpis***
–	Distal part of phallus with a weakly sclerotised, slice-shaped cornutus (Fig. 23)	***L. bipunctalis***

#### Loxoneptera hampsoni

Taxon classificationAnimaliaLepidopteraCrambidae

Chen & Zhang nom. nov.

15819497-8989-54E2-9077-4AED5CE0EC86

**Figures 2–9.**
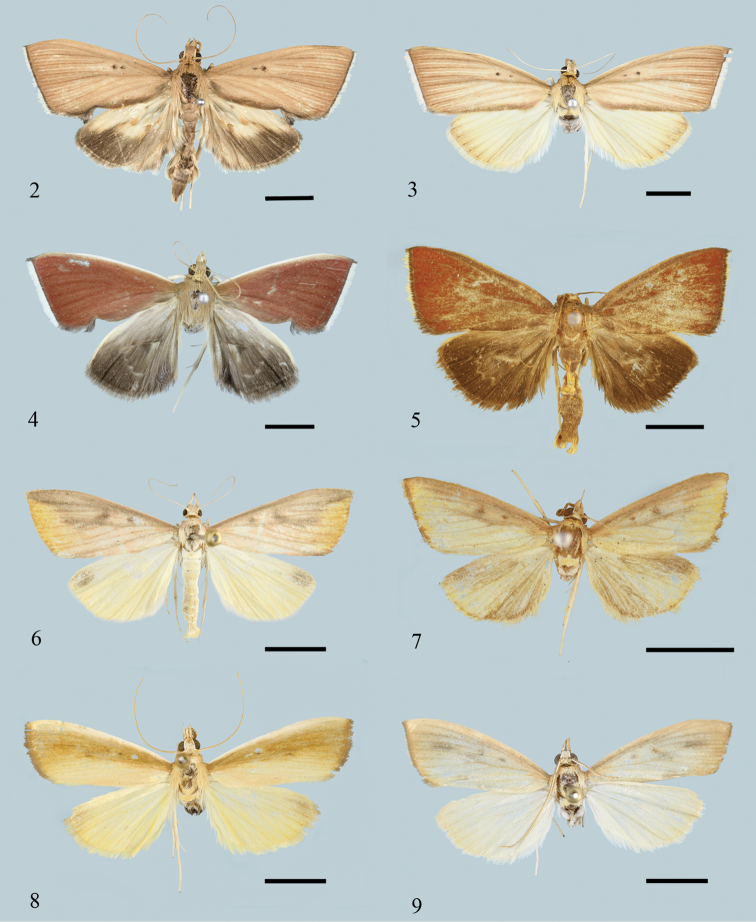
Adults of *Loxoneptera* spp. **2***L. hampsoni* nom. nov., male (Tibet) **3***L. hampsoni* nom. nov., female (Hainan) **4***L. albicostalis*, male (Yunnan) **5***L. crassiuncata* sp. nov., paratype, male (Yunnan) **6***L. carnealis*, male (Yunnan) **7***L. triangularis* sp. nov., holotype, male (Yunnan) **8***L. rectacerosa* sp. nov., holotype, male (Yunnan) **9***L. medialis*, male (Guangdong). Scale bars: 5.0 mm.

Figs 2 , 3 , 15 , 26

Loxoneptera carnealis Hampson, 1896: 406, fig. 219 (a junior secondary homonym of Notaspis carnealis Swinhoe, 1895). TL: India (Sikkim). TD: NHMUK.

##### Material examined.

***Type material*.** Type ♂, Sikkim, O. Müller [Coll.], Pyralidae Brit. Mus. Slide No. 9752 (NHMUK).

**Figures 10–13.**
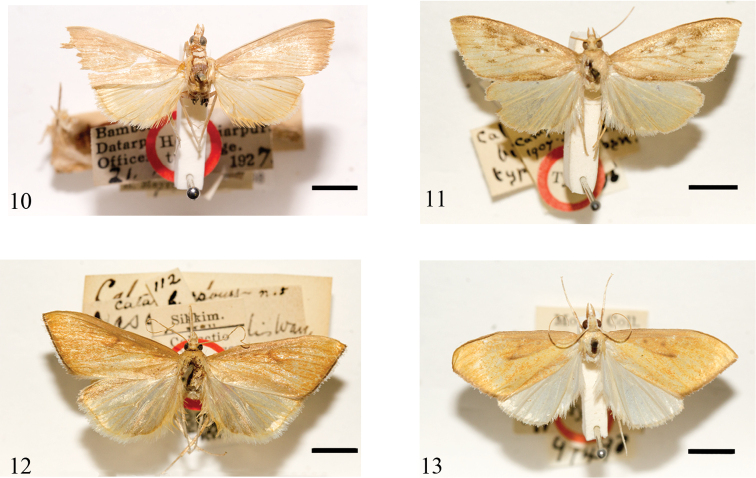
Adults of *Loxoneptera* spp. **10***L. pentasaris*, holotype, male (India) **11***L. bipunctalis*, type, male (India) **12***L. brevipalpis*, holotype, male (India) **13***L. dichroma*, type, male (India). Scale bars: 5.0 mm.

***Other material examined*. China. Hainan**: 1♂, Mt. Limushan, 5.V.2011, leg. Zhang Dandan & Yang Lijun; 1♂1♀, Mt. Limushan, 6.V.2011, leg. Zhang Dandan & Yang Lifeng, genitalia slide no. SYSU0117 (♂), no. SYSU0130 (♀); 1♂1♀, Mt. Limushan, 19.17°N, 109.73°E, alt. 662 m, 20.V.2013, leg. Li Jinwei, genitalia slide no. SYSU0929 (♂), no. SYSU0991 (♀), molecular voucher no. LEP0166 (♂), no. LEP0174 (♀). **Yunnan**: 2♂, Mengla, Xishuangbanna, 4, 6.X.2004, leg. R. L. Kitching, genitalia slide no. FCEL0003 (FCEL). **Tibet**: 1♂, 80K, Medog County, 29.66°N, 95.49°E, alt. 2059 m, 8.VIII.2017, leg. Qi Mujie & Yang Xiaofei (NKU); 1♀, Beibeng Village, Medog County, 29.24°N, 95.17°E, alt. 987 m, 12.VIII.2017, leg. Qi Mujie & Yang Xiaofei (NKU); 1♀, Beibeng Village, Medog County, 29.25°N, 95.18°E, alt. 810 m, 15.VIII.2017, leg. Qi Mujie & Yang Xiaofei (NKU).

##### Diagnosis.

*Loxoneptera hampsoni* is easily distinguished from other *Loxoneptera* species as follows: forewing with distinct, black-brown and point-like orbicular and reniform stigmata, bearing pale yellow stripes between veins, and veins with ochre-brown scales forming streaks; dorsal sella with a long and slender rod-shaped extension in the male genitalia.

##### Redescription.

***Head*.** Frons brown, with white lateral bands. Vertex brown, mixed with some white erected scales. Labial palpus dark brown, with white scales on ventral side. Maxillary palpus brown. Antennae brown. ***Thorax*.** Dorsal side, patagia and tegula yellowish brown, ventral side grey white. Foreleg yellowish brown, dorsal tarsus grey white; ventral femur and tibia of midleg and hindleg grey white, others pale yellow. ***Wings*.** Wingspan 29.0–36.0 mm. Forewing termen straight, a small triangular indentation presented on 1/3 of posterior margin in male, and with a group of black-brown scales; yellowish brown, mixed with ochre-brown scales, pale yellow stripes presented between veins, and veins covered with ochre-brown scales forming streaks; orbicular stigma appearing as a black point, reniform stigma black, small and round; fringe white, basal 1/5 black-brown. Hindwing in male black-brown on terminal area, remaining areas pale yellow, a triangular patch present near posterior angle of cell, slightly concave and densely covered with pale brown scales; in female pale yellow, mixed with ochre-brown scales on termen; fringe brown in male, pale yellow in female. ***Abdomen*.** Dorsal side of abdomen black-brown, ventral side grey white; 5^th^ abdominal segment with a group of pale yellow scales on each side in male; sternite VIII in male slightly sclerotised with two pointed anterolateral processes.

***Male genitalia*** (Fig. 15). Uncus somewhat wide and short, distally narrowly rounded, without setae. Saccus narrow. Dorsal projection of transtilla relatively thick and slightly curved, ~ 1/2 length of costa, distally bearing hair almost as long as projection. Valva with dorsal margin slightly concave, ventral margin nearly paralleled with dorsal margin, apex truncate; costa wide; dorsal sella membranous, rod-shaped, rather slender, and fragile; ventral sella sclerotised, with a somewhat straight, hook-shaped process; dorso-distal sella with a pointed process extended beyond ventral margin of valva; sacculus broad. Juxta heart-shaped, middle part concave inwardly, with wide arms. Phallus with vesica bearing two groups of spine-shaped cornuti, one longer and curved, another short and straight.

**Figures 14–19.**
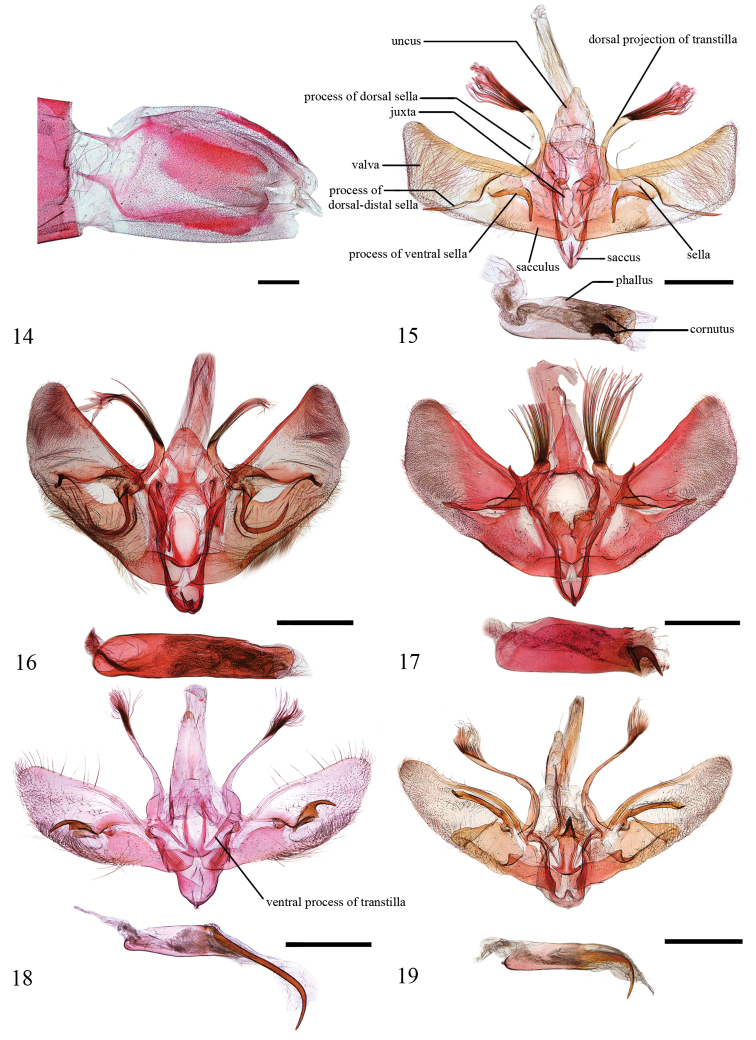
**14** The sternite VIII in male of *L. albicostalis***15–19** Male genitalia of *Loxoneptera* spp. **15***L. hampsoni* nom. nov., Hainan (genitalia slide no. SYSU0929) **16***L. albicostalis*, Yunnan (genitalia slide no. ZDD12108) **17***L. crassiuncata* sp. nov., Yunnan (genitalia slide no. FCEL0010) **18***L. carnealis*, Guizhou (genitalia slide no. SYSU0165) **19***L. triangularis* sp. nov., Yunnan (genitalia slide no. FCEL0004). Scale bars: 1.0 mm.

**Figures 20–25.**
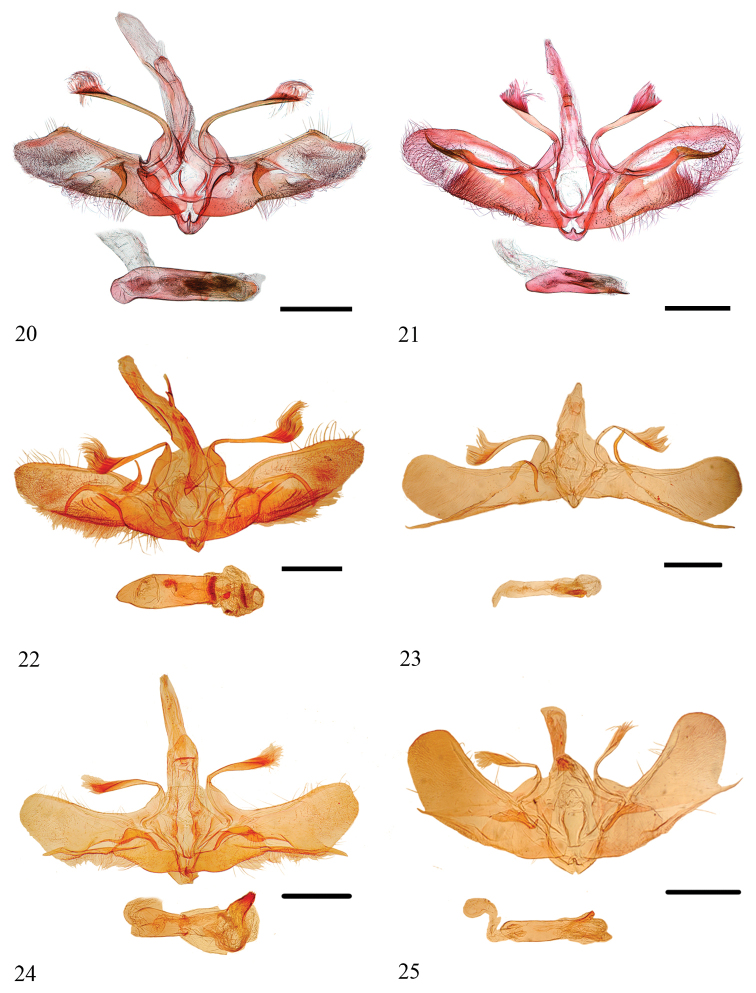
Male genitalia of *Loxoneptera* spp. **20***L. rectacerosa* sp. nov., Yunnan (genitalia slide no. ZDD12059) **21***L. medialis*, Guangdong (genitalia slide no. SYSU0987) **22***L. pentasaris*, India (Pyralidae Brit. Mus. Slide No. 9747) **23***L. bipunctalis*, India (Pyralidae Brit. Mus. Slide No. 9750) **24***L. brevipalpis*, India (Pyralidae Brit. Mus. Slide No. 9748) **25***L. dichroma*, India (Pyralidae Brit. Mus. Slide No. 9749). Scale bars: 1.0 mm.

**Figures 26–28.**
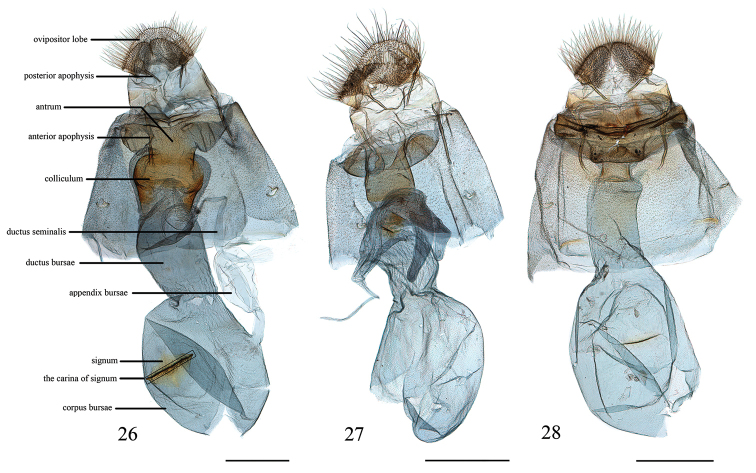
Female genitalia of *Loxoneptera* spp. **26***L. hampsoni* nom. nov., Hainan (genitalia slide no. SYSU0991) **27***L. carnealis*, Yunnan (genitalia slide no. SYSU0985) **28***L. medialis*, Guangdong (genitalia slide no. SYSU0990). Scale bars: 1.0 mm.

***Female genitalia*** (Fig. 26). Anterior apophyses 1.5 × as long as posterior apophyses. Lamella postvaginalis with weakly sclerotised transversely wrinkles, with dense and tiny spines; lamella antevaginalis with two curved and sclerotised notches. Antrum weakly sclerotised, cup-shaped, width 3 × as long as length; colliculum well developed and heavily sclerotised, expanded in middle part, length of colliculum ~ 1/3 of ductus bursae; ductus bursae slightly longer than length of corpus bursae; corpus bursae oval, appendix bursae arising from lateral side, small; signum broadly rhomboid, maximal length less than half width of corpus bursae, carina well-developed, laterally bearing with dense tiny spines, other two arms short and stout.

##### Distribution.

China (Hainan, Yunnan, Tibet), India.

##### Etymology.

The species is renamed after the last name of George Hampson, who proposed the genus *Loxoneptera* in 1896.

##### Remarks.

According to the characters of the male and female genitalia, *Calamochrous carnealis* (Swinhoe, 1895) is transferred to *Loxoneptera* in this paper, which creates a secondary homonym of *Loxoneptera carnealis* Hampson, 1896, the type species of *Loxoneptera*. The specific name of *Loxoneptera carnealis* Hampson, 1896 is not valid, therefore we give it a new replacement name, i.e., *Loxoneptera hampsoni* nom. nov.

#### Loxoneptera albicostalis

Taxon classificationAnimaliaLepidopteraCrambidae

Swinhoe, 1906

D5A63EF2-A8F5-53A2-B957-55E3F89B5BA6

Figs 4 , 14 , 16

Loxoneptera albicostalis Swinhoe, 1906: 415.

##### Material examined.

***Type material*.** Type ♂, Padang, W. Sumatra, Pyralidae Brit. Mus. Slide No. 9753 (NHMUK).

***Other material examined*. China. Yunnan**: 1♂, Jingpo Village, Nabang, Yingjiang Country, 24.71°N, 97.39°E, alt. 231 m, 2.VIII.2013, leg. Teng Kaijian et al., genitalia slide no. ZDD12108 (NKU).

##### Diagnosis.

In appearance, *Loxoneptera albicostalis* is extremely similar to *L. crassiuncata* and *Eumorphobotys horakae* Chen & Zhang, 2018 in the wing shape, the clean reddish brown forewing and the dark brown hindwing, but can be distinguished by the whiter costa of both wings, a group of dark brown scales on the posterior margin of forewing, and a group of scales on each side of the 5^th^ abdominal segment in male. The underside of forewing in *L. albicostalis* is smoky brown, while that of *E. horakae* is pale yellow from anterior margin of cell to posterior margin. The male genitalia resemble that of *L. crassiuncata* but can be differentiated by the shorter and stouter uncus, the relatively longer and slender dorsal projection of transtilla, nearly triangular dorsal sella, the long and hook-shaped process of the ventral sella, as well as the absence of the spine-shaped cornutus in phallus.

##### Redescription.

***Head*.** Frons brown. Vertex brown, mixed with some yellow erected scales. Labial palpus dark brown, with white scales on ventral side. Maxillary palpus brown. Antennae yellowish brown. ***Thorax*.** Dorsal side, patagia and tegula brown, ventral side grey white. Legs pale yellow to grey white; hindleg basal outer spur 1/5 of basal inner spur. ***Wings*.** Wingspan 32.0–36.0 mm. Forewing wide, reddish brown, without pattern; costal area white, mixed with pale brown scales at apex; termen straight; a small triangular indentation presented on the 1/3 of posterior margin in male, and with a group of black-brown scales; fringe white, with basal 1/4 black-brown. Hindwing black-brown, costa area pale yellow; a triangular patch presented near the posterior angle of cell, densely covered with pale brown scales; fringe black-brown. Underside smoky dark brown on forewing, and pale brown on posterior margin area. ***Abdomen*.** Dorsal side black-brown, ventral side grey white; abdominal segment V with a group of dark scales on each side in male; sternite VIII in male slightly sclerotised with two pointed anterolateral processes.

***Male genitalia*** (Fig. 16). Uncus wide and short, distally broadly rounded, with few hair-like setae. Saccus rounded. Dorsal projection of transtilla relatively thick and slightly curved, ~ 1/3 length of costa, distally bearing hairs longer than projection. Valvae with dorsal margin slightly straight, ventral margin sinuated, apex narrowly rounded; costa narrow; dorsal sella membranous with several setae, nearly triangular; ventral sella with a long, hook-shaped, and sclerotised process; dorso-distal sella bearing a short sclerotised process distally; sacculus broad, extended dorsad a triangular protrusion in middle. Juxta shield-shaped, middle part weakly sclerotised. Phallus slowly narrow to end, vesica mostly granulated.

***Female genitalia*.** Unknown.

##### Distribution.

China (Yunnan), Indonesia (Sumatra), Malaysia.

##### Remarks.

The forewing colour of the type material of *Loxoneptera albicostalis* is pale yellow tinged with some reddish brown scales and differs from the specimen collected in China. No obvious difference could be found in the male genitalia between the type specimen and the Chinese specimen.

#### Loxoneptera crassiuncata

Taxon classificationAnimaliaLepidopteraCrambidae

Chen & Zhang sp. nov.

4B680204-9A1C-569F-AE2A-37B79E2C187C

http://zoobank.org/E03FEF8B-F4B4-4DCD-9192-5971917D4729

Figs 5 , 17

##### Material examined.

***Type material*.** Holotype, ♂, **China: Yunnan**: Mengla, Xishuangbanna, 4.IX.2004, leg. R. L. Kitching, genitalia slide no. FCEL0010 (FCEL). Paratypes: **China: Yunnan**: 1♂, Mengla, Xishuangbanna, 28.IX.2004, leg. R. L. Kitching; 1♂, Mengla, Xishuangbanna, 29.IX.2004, leg. R. L. Kitching, genitalia slide no. FCEL0012 (FCEL).

##### Diagnosis.

*Loxoneptera crassiuncata* is similar to *L. albicostalis* in reddish brown forewing colour but male specimens can be distinguished by the unbroken posterior margin of forewing (without a small triangular indentation), without a group of black-brown scales, and abdominal segment V without a group of dark scales. In the male genitalia, it can be differentiated by the longer and slender uncus, the shorter and stouter dorsal projection of transtilla, the slender and rod-shaped dorsal sella, the relatively shorter and slightly curved process of the ventral sella, as well as the presence of a horn-shaped cornutus in phallus.

##### Description.

***Head*.** Frons brown. Vertex brown. Labial palpus brown, with white scales on ventral side. Maxillary palpus brown, broadened distally with scales. Antennae dark brown. ***Thorax*.** Dorsal side, patagia and tegula brown, ventral side grey white. Legs yellowish white or pale yellow, dorsal of midlegs and hindlegs yellowish brown; hindleg with basal outer spur 1/4 of inner spur. ***Wings*.** Wingspan 29.0–31.0 mm. Forewing wide, termen nearly straight; reddish brown, brown at basal half of posterior portion, costal band brown, without pattern; fringe pale yellow, basal half and the posterior angle black-brown. Underside greyish brown. Hindwing black-brown, pale yellow on anterior margin; a triangular patch presented near the posterior angle of cell, the margin of triangular patch with pale yellow scales and the outer margin dentate; fringe black-brown. Underside greyish brown. ***Abdomen*.** Dorsal side of abdomen brown, ventral side pale yellow; sternite VIII in male slightly sclerotised with two pointed anterolateral processes.

***Male genitalia*** (Fig. 17). Uncus slightly narrow, distally narrowly rounded, with several setae. Saccus narrow. Dorsal projection of transtilla rather thick and straight, ~ 1/4 length of costa, distally bearing setae ~ 2 × length of projection. Valva with dorsal margin slightly concave, ventral margin nearly parallel with dorsal margin, and apex slightly truncate; costa narrow; dorsal sella membranous, long and slender, rod-shaped and fragile; ventral sella with a stick-like and strongly sclerotised process; dorso-distal sella with a short stick-like process, pointed apically; sacculus broad, extended dorsad with a triangular protrusion in the middle. Juxta with basal part narrow, two arms rather broad. Phallus stout, vesica with a horn-shaped and strongly sclerotised cornutus apically.

***Female genitalia*.** Unknown.

##### Distribution.

China (Yunnan).

##### Etymology.

The specific name is derived from the Latin *crassi*- (thick) and *uncatus* (horn-shaped), referring to the shape of cornuti in the phallus.

#### Loxoneptera carnealis

Taxon classificationAnimaliaLepidopteraCrambidae

(Swinhoe, 1895) comb. nov.

F147A604-6A7F-54A4-9CB4-36F83F24149D

Figs 6 , 18 , 27

Notaspis carnealis Swinhoe, 1895: 302.
Calamochrous carnealis (Swinhoe): Hampson, 1896: 420.

##### Material examined.

***Type material*.** Type ♂, Khasi Hills., 95-224, Cherra Punji (NHMUK). Syntype: 1♂, Cherra Punji, Swinhoe Coll., Brit. Mus. 1926-239 (NHMUK).

***Other material examined*. China. Guangdong**: 6♂, Shimentai Reserve, Yingde, 27.V.2012, leg. Yang Lijun & Jia Qianju. **Guizhou**: 1♂, Maolan Reserve, 25.13°N, 107.87°E, alt. 797 m, 12.VII.2013, leg. Chen Xiaohua, genitalia slide no. SYSU0165, molecular voucher no. LEP0044; 1♀, Banzhai Village, Maolan Reserve, 25.23°N, 108.03°E, alt. 530 m, 11.VIII.2018, leg. Zheng Meiling et al. (NKU). **Yunnan**: 6♂, Baihualing Reserve, Baoshan, alt. 1520 m, 12–13.VIII.2007, leg. Zhang Dandan, genitalia slide no. LJW12064, no. LJW12098; 2♂1♀, Tropical Botanical Garden, Xishuangbanna, 21.92°N, 101.27°E, alt. 606 m, 22.XI.2017, leg. Chen Kai & Liu Qingming, genitalia slide no. SYSU0986 (♂), no. SYSU0985 (♀), molecular voucher no. LEP0186; 1♂, Tropical Botanical Garden, Xishuangbanna, alt. 550 m, 13.III.2014, leg. Zhang Zhenguo, molecular voucher no. LEP0169 (NKU); 2♂, Mengla, 1000 m, 11–12.VII.2012, leg. Kitching & Ashton, genitalia slide no. FCEL0005 (FCEL).

##### Diagnosis.

This species is similar to *Loxoneptera triangularis* in appearance, but can be distinguished by the following characters: forewing mixed with reddish brown scales, a distinct dark brown stripe appearing near posterior angle of cell; apex of hindwing with a dark brown patch; dorso-distal sella with a hook-shaped process; distal end of phallus with a spine-shaped process, longer than phallus length.

##### Redescription.

***Head*.** Frons pale reddish brown, with white lateral bands. Vertex pale brown, mixed with some reddish brown erect scales. Labial palpus reddish brown, with white scales on ventral side. Maxillary palpus reddish brown, broadened distally with scales. Antennae yellowish brown. ***Thorax*.** Dorsal patagia and tegula ochre-brown, ventral side grey-white. Legs pale yellow to grey-white; hindleg basal outer spur 2/5 of basal inner spur. ***Wings*.** Wingspan 22.0–29.0 mm. Forewing yellowish brown, densely mixed with reddish brown scales; dark brown from costal margin to posterior margin of cell; costal margin white; orbicular stigma appearing as a black-brown point, reniform stigma black, appearing as a thick streak on discocellulars; a distinct dark brown stripe appearing near posterior angle of cell; fringe black-brown. Hindwing pale yellow, apex with a dark brown patch; fringe pale yellow. Underside of forewing pale yellow, black-brown from costal margin to posterior margin of cell. ***Abdomen*.** Dorsal side of abdomen black-brown, ventral side grey white; sternite VIII in male slightly sclerotised with two pointed anterolateral processes.

***Male genitalia*** (Fig. 18). Uncus long, triangular, distally narrowly rounded, with few hair-like setae. Saccus rounded. Dorsal projection of transtilla relatively slender and slightly curved, ~ 3/4 length of costa, distally bearing hair ~ 1/2 length of projection, basal 1/3 broad. Valva with dorsal margin slightly convex, ventral margin sinuated, apex narrowly rounded; costa narrow; dorsal sella membranous with several setae, nearly rectangular; ventral sella with short, finger-shaped, and weakly sclerotised process; dorso-distal sella bearing a hook-shaped, strongly sclerotised process, basal broad with two small spins; sacculus broad. Juxta with basal part narrow, two arms long and slender, pointed apically. Phallus short, distal part with a long and pointed spine, slightly curved, as long as the length of phallus.

***Female genitalia*** (Fig. 27). Anterior apophyses 1.5 × as long as posterior apophyses. Antrum weakly sclerotised, cup-shaped; colliculum well developed, length of colliculum ~ 2/7 of ductus bursae; basal ductus seminalis expanded and sclerotised; ductus bursae short and stout, as long as length of corpus bursae; corpus bursae oval, without appendix bursae and signum.

##### Distribution.

China (Guangdong, Guizhou, Yunnan), India.

#### Loxoneptera triangularis

Taxon classificationAnimaliaLepidopteraCrambidae

Chen & Zhang sp. nov.

ADD4EE3D-5F18-5D86-9E2F-10ACFB8DB4DF

http://zoobank.org/A58926C8-3A03-4017-89BB-F613163F7686

Figs 7 , 19

##### Material examined.

***Type material*.** Holotype, ♂, **China: Yunnan**: Mengla, Xishuangbanna, 7.X.2004, leg. R. L. Kitching, genitalia slide no. FCEL0004 (FCEL). Paratype: **China: Yunnan**: 1♂, Mengla, Xishuangbanna, 4.X.2004, leg. R. L. Kitching.

##### Diagnosis.

Externally, *Loxoneptera triangularis* resembles *L. carnealis* in the wing shape, but can be distinguish by the smaller wings, and costal and posterior areas of hindwing dark brown. In the male genitalia, it can be differentiated by the process on the dorso-distal sella with a strongly sclerotised stick, distal part of juxta with a strongly sclerotised and narrowly triangular process, and distal phallus with a relatively short and hook-shaped spine.

##### Description.

***Head*.** Frons pale yellow, with white lateral bands, basal white bands mixed with reddish brown scales. Vertex pale yellow. Labial palpus reddish brown, ventral side with white scales. Maxillary palpus reddish brown, broadened distally with scales. Antennae yellowish brown. ***Thorax*.** Dorsal side, patagia and tegula yellowish brown, mixed with reddish brown scales, ventral side grey white. Legs pale yellow. ***Wings*.** Wingspan 23.0–25.0 mm. Forewing pale yellow, termen dark brown, as well as from costal margin to posterior margin of cell, apex mixed with reddish brown scales; orbicular stigma weak, appearing as a dark brown point, reniform stigma black-brown and weak; fringe dark brown. Underside of forewing black from costal margin to posterior margin of cell. Hindwing pale yellow between CuA_2_ and M_2_, remainders dark brown, without pattern, fringe yellow brown. ***Abdomen*.** Dorsal side of abdomen pale brown, ventral side grey white; sternite VIII in male slightly sclerotised with two stout anterolateral processes.

***Male genitalia*** (Fig. 19). Uncus long and slender, distally narrowly rounded, with few hair-like setae. Saccus rounded. Dorsal projection of transtilla relatively slender and slightly curved, approximately as long as length of costa, distally bearing hair ~ 1/4 length of projection, basal 1/3 broad. Valva with dorsal margin slightly convex, ventral margin sinuated, apex narrowly rounded; costa slightly curved; dorsal sella membranous, with several setae, ventral sella with a small, hook-shaped and sclerotised process, narrow and pointed apically; dorso-distal sella with a long, stick-like, strongly sclerotised process, broad at terminal part, then pointed at apex; sacculus broad. Juxta shield-shaped, strongly sclerotised, distal part broad, with a strongly sclerotised and narrowly triangular process. Phallus long, distal end with a long and hook-shaped spine, narrow and pointed apically.

***Female genitalia.*** Unknown.

##### Distribution.

China (Yunnan).

##### Etymology.

The specific name derived from the Latin *triangularis*, referring to the triangular process in the end of juxta.

#### Loxoneptera rectacerosa

Taxon classificationAnimaliaLepidopteraCrambidae

Chen & Zhang sp. nov.

F07D23B7-0137-5E93-84BB-6ED94C44135A

http://zoobank.org/FF3B831C-02BE-407A-ABC2-F49CAEEBE759

Figs 8 , 20

##### Material examined.

***Type material*.** Holotype, ♂, **China: Yunnan**: Yexianggu, Xishuangbanna, 22.17°N, 100.87°E, alt. 762 m, 18.VII.2014, leg. Teng Kaijian et al., genitalia slide no. ZDD12059, molecular voucher no. LEP0170 (NKU).

##### Diagnosis.

*Loxoneptera rectacerosa* resembles *L. medialis* in wing pattern, but the forewing of *L. rectacerosa* is brown from the costal margin to posterior margin of the cell, and white on costal margin, whereas it is pale yellow in *L. medialis*. In the male genitalia, dorsal margin of valva of *L. rectacerosa* makes a turn in the end, forming a distinct obtuse subapical angle; the process of the dorso-distal sella is smaller and shorter than that of *L. medialis*; distal end of phallus has a small and triangular sclerite, vesica is just with a group of spines.

##### Description.

***Head*.** Frons pale yellow, with white lateral bands. Vertex pale yellow. Labial palpus brown, with white scales on ventral side. Maxillary palpus brown, broadened distally with scales. Antennae yellowish brown. ***Thorax*.** Dorsal side, patagia and tegula yellowish brown, ventral side grey white. Legs white to yellowish white. ***Wings*.** Wingspan 29.0 mm. Forewing brown, mixed with reddish brown scales, costal margin white, posterior area pale yellow; orbicular stigma weak, appearing as a dark brown point, reniform stigma absent; fringe black-brown. Hindwing pale yellow, without any spot, apex mixed with a few pale brown scales. Underside of forewing black on cell. ***Abdomen*.** Dorsal side of abdomen pale brown, ventral side grey white; sternite VIII in male slightly sclerotised with two stout anterolateral processes.

***Male genitalia*** (Fig. 20). Uncus long and slender, distally narrowly rounded, with few hair-like setae. Saccus rounded. Dorsal projection of transtilla relatively slender and slightly curved, ~ as long as length of costa, distally bearing hair ~ 1/4 length of projection, basal 1/3 broad. Valva with dorsal margin slightly convex, ventral margin sinuated, apex slightly pointed; costa straight, and making a turn on 1/5 of the end, forming a break angle on dorsal margin of valva subapically; dorsal sella membranous, with several setae; ventral sella with a hook-shaped and strongly sclerotised process, narrow and pointed apically; dorso-distal sella with a short and weakly sclerotised process; sacculus broad. Juxta with basal part narrow, two arms long and slender, pointed apically. Phallus long and slightly curved, distal end with a semi-circular sclerite, vesica with a group of short, straight, spine-shaped cornuti.

***Female genitalia*.** Unknown.

##### Distribution.

China (Yunnan).

##### Etymology.

The specific name derived from the Latin *rect*- (straight) and *arcerosus* (spine-shaped), referring to the shape of cornuti in phallus.

#### Loxoneptera medialis

Taxon classificationAnimaliaLepidopteraCrambidae

(Caradja, 1925) comb. nov.

F254E73D-7594-586A-BCE8-A62D5880F457

Figs 9 , 21 , 28

Calamochrous medialis Caradja, 1925: 363.

##### Material examined.

***Type material*.** Holotype, ♂, Canton, Type, Car.[adja], Gen. Praep.[Prep.] EGM 3 (MGAB).

***Other material examined*. China. Guangdong**: 1♂, Dongmei Village, Potou District, Zhanjiang, 10.IV.2016, leg. Li Zhiqiang & Li Jun, genitalia slide no. SYSU0987, molecular voucher no. LEP0171; 1♀, Liuzhang Village, Beihe Country, Leizhou, 9.IV.2016, leg. Li Zhiqiang & Li Jun, genitalia slide no. SYSU0990, molecular voucher no. LEP0173. **Hainan**: 1♂, Jianling Reserve, 18.87°N, 110.27°E, alt. 143 m, 8.IX.2013, leg. Chen Xiaohua, genitalia slide no. SYSU0180, molecular voucher no. LEP0096.

##### Diagnosis.

The wing shape of *Loxoneptera medialis* is similar to *L. rectacerosa* but can be distinguished by the light yellow forewing and costal margin. In the male genitalia, it can be distinguished by longer spinous process on dorso-distal sella, distal end of phallus with a small and pointed spine, and vesica with two groups of short, spine-shaped cornuti.

##### Redescription.

***Head*.** Frons pale yellow, with white lateral bands. Vertex pale yellow. Labial palpus pale yellow, with white scales on ventral side. Maxillary palpus pale yellow, mixed with white scales, broadened distally with scales. Antennae yellowish brown. ***Thorax*.** Dorsal side, patagia and tegula yellowish brown, ventral side grey white. Legs yellowish white. ***Wings*.** Wingspan 25.0–30.0 mm. Forewing pale yellow, costal and terminal areas reddish brown; orbicular stigma weak, dark brown, reniform stigma weak, black-brown, appearing as a thick line on discocellulars; a weak, dark-brown stripe appearing between M_2_ and CuA_1_; fringe black-brown. Hindwing pale yellow, termen mixed with brown scales, without pattern. Underside of forewing pale yellow, without any spot. ***Abdomen*.** Dorsal side of abdomen black-brown, ventral side grey white; sternite VIII in male slightly sclerotised with bifurcate anterolateral processes.

***Male genitalia*** (Fig. 21). Uncus long and slender, distally narrowly rounded, with few hair-like setae. Saccus rounded. Dorsal projection of transtilla relatively slender and slightly curved, approximately as long as length of costa, distally bearing hair ~ 1/3 length of projection, basal 1/3 broad. Valva with dorsal margin slightly convex, ventral margin sinuated, apex slightly pointed; costa slightly curved; dorsal sella appearing as a broad, slightly curved and stick-like sclerite, with several setae; ventral sella sclerotised, with a long, straight and stick-like process, narrow and pointed apically, apex slightly curved; dorso-distal sella with a pointed, hook-like, and strongly sclerotised process, as long as the process on ventral sella. Sacculus broad. Juxta with basal part narrow, two arms long and slender, pointed apically. Phallus short, basal 1/2 broad, distal end with a small pointed spine, and vesica with two groups of short, spine-shaped cornuti.

***Female genitalia*** (Fig. 28). Anterior apophyses 1.5 × as long as posterior apophyses; lamella postvaginalis trapezoidal and strongly sclerotised, with distinct transversely wrinkles, covered with dense and tiny spines; lamella antevaginalis strongly sclerotised, appearing as a small, triangular sclerite, covered with many dense and tiny spines. Antrum strongly sclerotised, cup-shaped; colliculum well developed and strongly sclerotised; ductus bursae short and stout, ~ 1/2 length of corpus bursae; corpus bursae oval, signum weak, reduced into a long carina, laterally bearing with some tiny spines, without appendix bursae.

##### Distribution.

China (Guangdong, Hainan).

#### Loxoneptera pentasaris

Taxon classificationAnimaliaLepidopteraCrambidae

(Meyrick, 1932) comb. nov.

6277F985-99D8-5A1A-BD83-6C7BC89CD789

Figs 10 , 22

Calamochrous pentasaris Meyrick, 1932: 317.

##### Material examined.

***Type material*.** Holotype, ♂, [India] Datarpur, Hoshiarpur. Officer-in-charge, 21.12.1927, Pyralidae Brit. Mus. Slide No. 9747 (NHMUK).

##### Diagnosis.

Wingspan 28.0 mm. *Loxoneptera pentasaris* is best distinguished from other *Loxoneptera* species by greyish ochreous forewing with a white costal band, and without pattern. In the male genitalia, this species is similar to *L. medialis* in the shape of dorsal projection of transtilla, ventral sella and valva, but can be distinguished by the triangular dorsal sella, process of dorso-distal sella extending ventrad, distal margin of phallus densely decorated with short spines.

##### Distribution.

India.

#### Loxoneptera bipunctalis

Taxon classificationAnimaliaLepidopteraCrambidae

(Hampson, 1912) comb. nov.

2BDFF375-594C-56B1-9B99-468A5B401517

Figs 11 , 23

Calamochrous bipunctalis Hampson, 1912: 1269.

##### Material examined.

***Type material*.** Type ♂, S. India, Palani Hills [Palnis], Campbell 1907.365, Pyralidae Brit. Mus. Slide No. 9750 (NHMUK).

##### Diagnosis.

Wingspan 34.0 mm. In appearance, *Loxoneptera bipunctalis* is best distinguished from other *Loxoneptera* species by pale ochreous yellow forewing, two blackish orbicular stigmata, and interrupted postmedial line of forewing. In the male genitalia, this species is similar to *L. brevipalpis* and *L. dichroma* but can be distinguished by the longer process of dorso-distal sella and the weakly sclerotised, slice-shaped cornutus of phallus.

##### Distribution.

India.

#### Loxoneptera brevipalpis

Taxon classificationAnimaliaLepidopteraCrambidae

(Snellen, 1890) comb. nov.

3801E359-ABF7-5384-974B-315EF91DAE7F

Figs 12 , 24

Calamochrous brevipalpis Snellen, 1890: 599.

##### Material examined.

***Type material*.** Holotype, ♂, Sikkim, O. Möller, Pyralidae Brit. Mus. Slide No. 9748 (NHMUK).

##### Diagnosis.

Wingspan 33.0 mm. This species is distinguished by dull luteous forewing suffused with ochreous scales and bearing indistinct orbicular and reniform stigmata, lustrous hindwing suffused with grey scales along the costa. In the male genitalia, this species is similar to *L. dichroma* in the shape of the dorsal projection of the transtilla, ventral sella and valva, as well as by the process of dorso-distal sella extended ventrad and beyond the ventral margin of valva. *Loxoneptera brevipalpis* can be distinguished by the thick and heavily sclerotised process of dorso-distal sella, and the heavily sclerotised, spiny, thumb-shaped cornutus.

##### Distribution.

India (Sikkim).

#### Loxoneptera dichroma

Taxon classificationAnimaliaLepidopteraCrambidae

(Moore, 1888) comb. nov.

5698B4B0-4927-5FC3-9D89-D6554AD7D214

Figs 13 , 25

Ebulea dichroma Moore, 1888: 223.
Calamochrous dichroma (Moore): Snellen, 1890: 599.
Anania dichroma (Moore): Nuss et al. 2003–2021, Global Information System on Pyraloidea.

##### Material examined.

***Type material*.** Type ♂, Darjeeling [Darjiling], Pyralidae Brit. Mus. Slide No. 9749 (NHMUK).

##### Diagnosis.

Wingspan 34.0 mm. This species can be distinguished by having a brown stripe along posterior margin of the discal cell in forewing, and ventral-distal wall of phallus is weakly sclerotised and obliquely extended into a process.

##### Distribution.

India.

## Discussion

The monophyly of *Loxoneptera* is strongly supported by the results of the molecular analysis. The dorsal projection of the transtilla in the male genitalia is a putative synapomorphy for the genus. It is shared by eleven species of *Loxoneptera* and can be used to separate them from most other pyraustine genera. In addition, two provisional infrageneric groups of the species of *Loxoneptera* are recognised by proportional lengths of the dorsal projection of the transtilla with its distal hair. The tree topology (Fig. 1) supports this morphological trait: *L. rectacerosa* is more closely related to *L. medialis* and *L. carnealis* (all bearing a relatively short hair, < 1/2 length of the dorsal projection of the transtilla) than to *L. albicostalis* and *L. hampsoni* (both bearing a relatively long hair, as long as or longer than the dorsal projection of the transtilla). Based on the proportional length of the dorsal projection of the transtilla with its distal hair, we additionally place *L. triangularis* and *L. crassiuncata*, as well as *L. pentasaris*, *L. bipunctalis*, *L. brevipalpis*, and *L. dichroma* (not included in the molecular analysis because no fresh specimen could be accessed) in their respective subgroups: *L. triangularis*, *L. bipunctalis*, *L. brevipalpis*, *L. dichroma*, and *L. pentasaris* resemble *L. rectacerosa*, *L. medialis*, and *L. carnealis* by the relatively short hair on the dorsal projection of the transtilla, while *L. crassiuncata* resembles *L. albicostalis* and *L. hampsoni* by the relatively long hair. The relationships among all these species need further study on more nucleotide sequences and freshly collected specimens.

After examining three specimens of *Calamochrous chilonalis* (the type species of *Calamochrous*) deposited in NHMUK, we confirm that it is quite different from those *Loxoneptera* species formerly placed in *Calamochrous*. Among these specimens, one is the paratype of which the abdomen was lost; the other two, collected in Minca, Colombia, were dissected and identified by Dr Koen V. N. Maes (Pyralidae Brit. Mus. Slide No. 762 male, 19967 female). Morphologically, *C. chilonalis* differs from *Loxoneptera* species by the relatively narrow forewing with arch termen, the conical, densely setose uncus, the narrowly triangular transtilla, the scale-like editum, as well as the long and slender ductus bursae. Maes (pers. comm.) also mentioned that *Calamochrous* species are restricted to the Nearctic region, while *Loxoneptera* species are distributed in the Oriental region.

## Supplementary Material

XML Treatment for Loxoneptera

XML Treatment for Loxoneptera hampsoni

XML Treatment for Loxoneptera albicostalis

XML Treatment for Loxoneptera crassiuncata

XML Treatment for Loxoneptera carnealis

XML Treatment for Loxoneptera triangularis

XML Treatment for Loxoneptera rectacerosa

XML Treatment for Loxoneptera medialis

XML Treatment for Loxoneptera pentasaris

XML Treatment for Loxoneptera bipunctalis

XML Treatment for Loxoneptera brevipalpis

XML Treatment for Loxoneptera dichroma
